# Recessive congenital methemoglobinemia: a systematic review of reported cases

**DOI:** 10.1186/s13023-026-04215-7

**Published:** 2026-02-04

**Authors:** Julie Neven, Tessi Beyltjens, Marije Meuwissen, Barbara De Bisschop, Jason Bouziotis, Machiel van den Akker

**Affiliations:** 1 Department of Pediatrics, University Hospital Antwerp, University of Antwerp, Drie Eikenstraat 655, Edegem, 2650 Belgium; 2 Center of Medical Genetics, University Hospital Antwerp/ University of Antwerp, Prins Boudewijnlaan 43, Edegem, 2650 Belgium; 3 Department of Neonatology, ZAS Middelheim Hospital, Lindendreef 1, Antwerp, 2020 Belgium; 4 Center for Medical Genetics Ghent, Ghent University Hospital, Ghent, 9000 Belgium; 5 Clinical Trial Center (CTC), CRC Antwerp, Antwerp University Hospital University of Antwerp, Edegem, Belgium; 6 Faculty of Medicine and Health Sciences, University of Antwerp, Antwerp, Belgium; 7 Department of Pediatric Hematology, ZAS Queen Paola Children’s Hospital, Lindendreef 1, Antwerp, 2020 Belgium; 8 Pediatric Hematology Oncology, University Hospital Antwerp, DrieEikenstraat 655, Edegem, Antwerp, 2650 Belgium

**Keywords:** Congenital methemoglobinemia, Cytochrome b5 reductase, *CYB5R* gene, Methylene blue

## Abstract

**Background:**

Recessive congenital methemoglobinemia (RCM) is a rare autosomal recessive disorder characterized by a deficiency of NADH-cytochrome b5 reductase. Under normal conditions, cytochrome b5 reductase and NADH reductase maintain methemoglobin in the physiological range by keeping heme iron in the Fe^2^^+^ state. RCM presents in two clinical forms. Type I presents with isolated cyanosis, while type II presents also with severe neurocognitive manifestations. Genetic analysis of the *CYB5R3* gene is often used for diagnosis. Treatment with methylene blue, ascorbic acid, or riboflavin can reduce cyanosis, but does not affect neurological outcomes.

**Main body:**

We conducted a systematic review of RCM, identifying all previously reported cases, including one from our center. In addition, we conducted a meta-analysis of individual participant data and provided a comprehensive review of clinical manifestations, genotype-phenotype correlations and treatment options, as well as proposing a diagnostic flow chart.

**Conclusion:**

In the case of RCM, diagnosis is primarily achieved through genetic analysis based on direct sequencing. The described pathogenic variants are specific to either type I or type II RCM when considered in combination with their allelic variants in compound heterozygous cases. Well known variants such as c.757G>A is associated with type I in both homo- and heterozygous state. Whereas c.226G> is associated with type I in compound heterozygous state with c.173G>A or c.827C>T and type 2 in homozygous state. Similar results are observed for multiple other variants. Ascorbic acid is the most commonly used treatment.

**Clinical trial number:**

Not applicable.

**Supplementary Information:**

The online version contains supplementary material available at 10.1186/s13023-026-04215-7.

## Background

Recessive congenital methemoglobinemia (RCM) is a rare autosomal recessive disorder characterized by NADH-cytochrome b5 reductase deficiency. Recent publication by Fan and Osawa (2025) provides a fascinating historical perspective, suggesting that congenital methemoglobinemia was accurately depicted in a 12th-century Japanese artwork, the Yamai no Sōshi. It places as a condition that has affected human populations for centuries, long before its biochemical basis was understood. RCM presents in two distinct clinical forms. Patients with type I RCM present with isolated cyanosis, while those with type II RCM also develop severe neurocognitive manifestations. Genetic analysis of the *CYB5R3* gene and/or enzyme analysis is often used for diagnosis. Treatment with methylene blue, ascorbic acid, or riboflavin can reduce cyanosis but does not improve neurological outcomes.

Methemoglobinemia is defined as the presence of more than 1% of methemoglobin (metHb) in the blood. It is characterized clinically by a decreased capacity of hemoglobin (Hb) to carry oxygen, resulting in cyanosis and hypoxia. MetHb contains iron in a ferric state (Fe^3+^) rather than the ferrous state (Fe^2+^) found in hemoglobin. Cytochrome b5 reductase and NADH reductase maintain heme iron in the ferrous state and keep metHb within the physiological range [1, 2]. Methemoglobinemia can be acquired or hereditary. The more common acquired form is caused by exposure to oxidising agents such as nitrite compounds, dapsone, and sodium chlorite [3, 4]. Congenital methemoglobinemia is most commonly caused by NADH-cytochrome b5 reductase deficiency (Online Mendelian Inheritance in Man (OMIM) #250800). This rare autosomal recessive disorder has an estimated prevalence of 1 per 100.000 [5] and is caused by biallelic pathogenic variants in the Cytochrome B5 Reductase 3 (*CYB5R3*) gene, which is located on chromosome 22q13.2.

Cytochrome b5 reductase is an NADH-dependent enzyme that exists in two forms: a soluble form and a membrane-bound form. The soluble form is found in the cytoplasm of circulating red blood cells and is involved in methemoglobin reduction. The enzyme catalyzes the transfer of electrons from the physiological electron donor NADH to the small hemoprotein of cytochrome b5. NADH facilitates the reduction of cytochrome b5, which can reduce the oxidized ferric ion of Hb, when an electron is transferred from reduced cytochrome b5 [2, 6]. This process converts ferric heme (Fe3+) back to the ferrous state (Fe2+). An alternative pathway for metHb reduction uses NADPH instead of NADH as an electron donor. NADPH is generated by glucose-6-phosphate dehydrogenase (G6PD). However, unlike cytochrome b5 for NADH, there is no physiological electron acceptor for NADPH. Therefore, this alternative pathway is not physiologically active. It is only activated when an extrinsic electron acceptor, such as methylene blue, is present. The inability of metHb to bind oxygen, coupled with the increased affinity of Fe^3+^ for oxygen, causes a left shift in the oxygen dissociation curve. This results in tissue hypoxia and functional anemia. Pathogenic *CYB5R3* variants that affect only the soluble form of cytochrome b5 reductase are associated with type I congenital methemoglobinemia.

The membrane-bound form of cytochrome b5 reductase is part of the microsomal electron transport system. It plays a role in various physiological processes, including cholesterol biosynthesis, fatty acid elongation, and desaturation [2]. Regulation of fatty acid chain elongation is particularly important for myelination and brain development [7]. Pathogenic *CYB5R3* variants that affect both the soluble and membrane-bound forms of cytochrome b5 reductase are associated with type II congenital methemoglobinemia.

In type I, the deficiency is restricted to the erythrocytes, causing well-tolerated methemoglobinemia. In type II, however, the deficiency affects erythrocytes, leukocytes, and virtually all body tissues, resulting in a severe neurological syndrome [2]. We conducted a systematic review of cases, case series, and reviews to improve our understanding of the clinical presentation, the relevance of genotype to phenotype in types I and II RCM, and diagnosis and treatment options.

## Materials and methods

### Systematic review

We conducted a systematic review of the literature in accordance with the Preferred Reporting Items for Systematic Reviews and Meta-Analyses (PRISMA) 2020 guidelines, with the aim of identifying all studies involving patients with recessive congenital methemoglobinemia.

### Inclusion and exclusion criteria

This retrospective study had no publication date or language restrictions. Data were gathered via a literature search of the *Pubmed* database using the keyword “congenital methemoglobinemia.” All relevant articles, including case reports, case series, and reviews, were examined. Additional cases were identified by consulting the references of the identified articles. Articles were selected for inclusion based on their titles and abstracts. Only cases reporting RCM based on bi-allelic (likely) pathogenic *CYB5R3* variants and/or low cytochrome b5 reductase activity according to local reference values were included. Articles were excluded if they were unrelated, if the full texts was unavailable, or if genetic and/or enzyme analysis confirming the diagnosis of RCM was absent.

### Study selection

One reviewer screened the titles and abstracts of all the articles from the search and references, determining which articles to review in full according to the inclusion and exclusion criteria set out in the protocol. All included articles were screened by two reviewers.

### Data collection

The following data were collected in a structured Excel extraction sheet (see Supplementary Table A): gender; consanguinity; origin; mutation analysis; type of genetic testing; age at onset; clinical manifestations at diagnosis; laboratory examinations (percentage of metHb; erythrocyte cytochrome b5 reductase activity in erythroid and/or non-erythroid cells); and treatment. If the molecular analysis was not performed, information on enzymatic activity was included. Data concerning gender, consanguinity, origin, mutation analysis, type of genetic testing, age at onset, clinical manifestations at diagnosis and laboratory examinations were extracted by two reviewers.

All *CYB5R3* variants are described according to the current Human Genome Variation Society nomenclature guidelines based on the NM_000398.7 (GRCh38) transcript (MANE transcript). Variants were also interpreted and classified according to the recommendations of the American College of Medical Genetics and Genomics recommendations [8].

### Statistical analysis

We conducted Individual Participant Data (IPD) meta-analyses for binary and continuous variables. Binary variables were analyzed using generalized estimating equations (GEE) with clustering by study, while continuous variables were analyzed using mixed-effects linear models with a random intercept at the study level. In cases of zero events in one RCM type (complete separation), we used Firth’s penalized logistic regression with cluster-level (study-level) bootstrapping to obtain bias-reduced estimates. RCM type was included as a fixed effect in all models. All analyses were conducted using StataNow/BE, version 19.

## Results and discussion

**Fig. 1:**
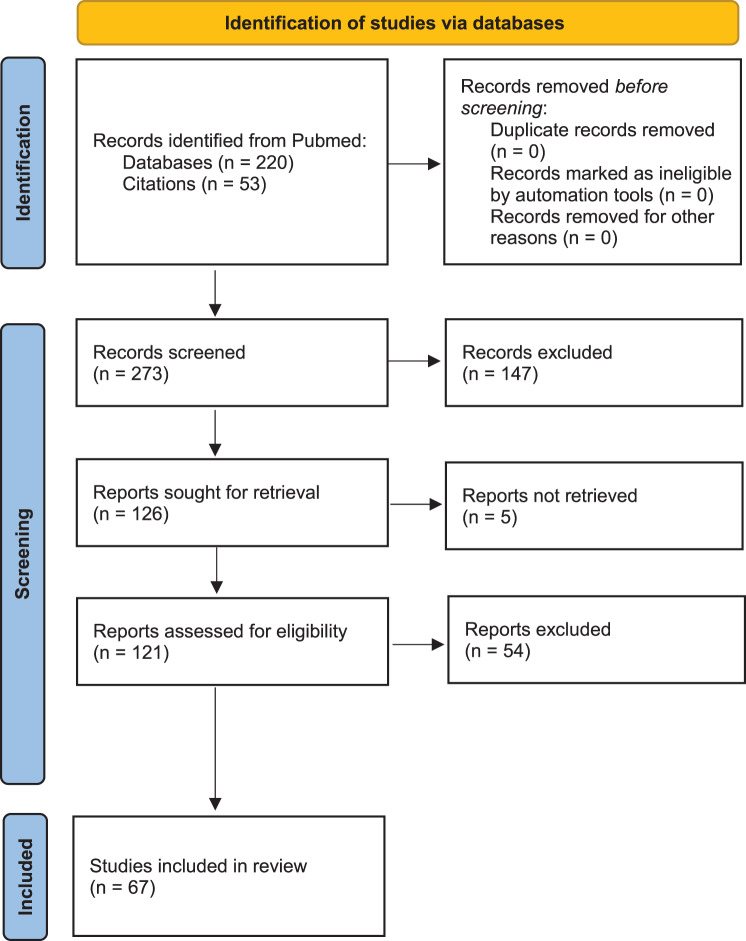
PRISMA flow diagram of the identification of the included articles

The search yielded 220 articles, of which 73 were selected based on their titles and/or abstracts. A further 53 articles were identified by consulting the references cited in these articles. Five reports could not be retrieved due to the full text being unavailable. Of the 121 articles reviewed, 97 described one or more cases of congenital methemoglobinemia. However, only those with confirmed genetic analysis and/or abnormal enzyme analysis were included, resulting in 67 articles being included in the final analysis. Each study reported between one and 38 cases (patients). In total, 205 cases were reported, including one new case from our centre. The publication dates of the included articles ranged from 1980 to 2024 (see Fig. 1). Information of the cases is summarized in Tables 1 (meta-analysis results of both binary and continuous variables), 2 (unique CYB5R3 variants), 3 (type of genetic analysis), and 4 (diagnostic tests and therapy). One supplementary table is available online: Table A (patient data). The results will be further elaborated in the discussion.

**Table 1:** Individual participant data (IPD) meta-analysis results of both binary and continuous variables

Variables	Type I			Type II			
	n	k	Estimate [95% CI]	n	k	Estimate [95% CI]	p-value
Consanguinity	57	19	0.72 [0.60 - 0.85]	42	21	0.63 [0.48 - 0.78]	0.34
Male	143	43	0.66 [0.58 - 0.74]	48	26	0.52 [0.38 - 0.66]	0.09
Female			0.34 [0.26 - 0.42]			0.48 [0.34 - 0.62]	
Age (years)	136	43	22.5 [17.8 - 27.2]	39	23	6.1 [0.2 - 12.1]	< 0.001
Vomiting	137	42	0.00 [0.00 - 0.01]	51	27	0.09 [0.00 - 0.19]	< 0.001
Seizures	137	42	0.01 [0.00 - 0.03]	51	27	0.12 [0.03 - 0.21]	0.01
Athetosis	137	42	0.00 [0.00 - 0.01]	51	27	0.22 [0.05 - 0.40]	< 0.001
Dystonia	137	42	0.00 [0.00 - 0.01]	51	27	0.34 [0.13 - 0.55]	< 0.001
Growth retardation/failure to thrive	137	42	0.00 [0.00 - 0.01]	51	27	0.32 [0.15 - 0.48]	< 0.001
Convergent squint/stabismus	137	42	0.00 [0.00 - 0.01]	51	27	0.36 [0.11 - 0.60]	< 0.001
Mental retardation	137	42	0.00 [0.00 - 0.01]	51	27	0.51 [0.29 - 0.73]	< 0.001
Spastic tetraparesis/hypertonia	137	42	0.00 [0.00 - 0.01]	51	27	0.41 [0.21 - 0.62]	< 0.001
Microcephaly	137	42	0.00 [0.00 - 0.01]	51	27	0.72 [0.56 - 0.89]	< 0.001
Global development delay	137	42	0.01 [0.00 - 0.02]	51	27	0.72 [0.60 - 0.85]	< 0.001
Dizziness/palpitations	137	42	0.02 [0.00 - 0.04]	51	27	0.01 [0.01 - 0.01]	0.38
Fatigue	137	42	0.30 [0.00 - 0.94]	51	27	0.01 [0.01 - 0.01]	0.01
Headache	137	42	0.30 [0.00 - 0.94]	51	27	0.01 [0.01 - 0.01]	0.01
Dyspnea	137	42	0.37 [0.00 - 0.81]	51	27	0.07 [0.00 - 0.21]	0.14
Hypotonia (esp. Axial)	137	42	0.00 [0.00 - 0.01]	51	27	0.24 [0.08 - 0.40]	< 0.001
Cyanosis	137	42	0.95 [0.90 - 1.00]	48	26	0.88 [0.78 - 0.98]	0.15
MetHb %	142	41	22.3 [19.4 - 25.3]	44	22	28.6 [24.5 - 32.6]	0.007
Riboflavin	32	22	0.07 [0.00 - 0.16]	14	12	0.07 [0.00 - 0.21]	0.97
Ascorbic ascid	32	22	0.86 [0.73 - 0.99]	14	12	0.93 [0.80 - 1.00]	0.51
Methylen blue	32	22	0.40 [0.20 - 0.59]	14	12	0.15 [0.00 - 0.34]	0.12
Erythrocyte metHb RA (U/g Hb)	80	23	7.17 [4.74 - 9.60]	30	14	4.02 [1.39 - 6.65]	< 0.001
Low erythrocyte metHb RA	88	29	0.97 [0.92 - 1.00]	41	20	0.99 [0.98 - 0.99]	0.38

Data presented as estimated probability or estimated mean with 95% confidence interval *n* = number of observations; k = number of studies; RA = reductase activity

### Patient characteristics

These are provided in Table 1. Information on sex was available for 143 cases of type I RCM and 48 cases of type II RCM. A total of 119 patients were male and 72 cases were female. The majority of patients were male in both types of RCM: 66% ([95% CI 58%-74%]) and 52% ([95% CI 38%-66%]) respectively. The mean age at presentation was significantly lower for type II RCM than for type I: 6 years [95% CI 0.2–12.1] and 22 years and 6 months [95% CI 17.8–27.2] respectively (*p* < 0.001). The younger age of type II patients could be explained by more prominent clinical features (see below) compared to the more subtle cyanosis of type I RCM patients, resulting in a later presentation. As RCM is a recessive disease, consanguinity was reported in many cases (type I 72% [95% CI 60%-85%]; type II 63% [95% CI 48%-78%]), and there was no statistically significant difference between the two types (*p* = 0.34).

### Clinical manifestations

The autosomal recessive form of methemoglobinemia consists of two clinical phenotypes. Type I is characterized by central cyanosis that does not improve with oxygen therapy and is usually present at birth [2, 9, 10]. Later in life, type I can be associated with exertional dyspnea, chest pain, headaches, and/or fatigue. The severity depends on the level and rate of metHb increase, as well as the patient’s underlying functional status. Mild compensatory erythrocytosis is often present; this is a physiological process that prevents clinically significant tissue hypoxia [11]. While RCM type I is a benign disorder with normal life expectancy [12], RCM type II has a more severe clinical course. It is stated in the literature that RCM type II constitutes approximately 10% of all RCM cases [13]. However, this review found that 25% of reported cases exhibited a RCM type II phenotype. This higher percentage could be due to underreporting of type I in the literature because of its benign clinical course. The full clinical manifestations of type II RCM usually manifests at 6–9 months. In addition to the characteristic cyanosis, other common features reported in the literature include progressive microcephaly, mild to severe impairment of brain development resulting in intellectual disability, delayed growth, opisthotonus, generalized hypertonia, dystonia or hypotonia, axial hypotonia, and movement disorders [2, 3, 14]. These symptoms are detailed in Table 1. Clinical symptoms were reported for 137 cases of type I RCM and 51 cases of type II RCM. The main symptoms of type I RCM are cyanosis (95% [95% CI 90%-100%]), fatigue (30% [95% CI 0%-94%]), headache (30% [95% CI 0%-94%]) and dyspnea (37% [95% CI 0%-81%]). In type II RCM, we estimated cyanosis as one of the presenting symptoms in approximately 88% [95% CI 78%-98%]) of cases. Additionally, the following symptoms are commonly reported: global developmental delay (72% [95% CI 60%-85%]), microcephaly (72% [95% CI 56%-89%]), intellectual disability (51% [95% CI 29%-73%]), spastic tetraparesis/hypertonia (41% [95% CI 21%-62%]), hypotonia (predominantly axial) (24% CI [95% CI 8%-40%]), dystonia (34% [95% CI 13%-55%]), failure to thrive (32% CI [95% CI 13%-48%]) and convergent squint or strabismus (36% [95% CI 11%-60%]). These symptoms were significantly associated with type II RCM (*p* < 0.001). Less frequent findings include epilepsy, chorea-athetotic or dyskinetic movement disorders, and feeding difficulties. Due to progressive neurological deterioration and associated complications, life expectancy is significantly reduced. Death is to be expected in the first decade of life, predominantly due to swallowing difficulties and respiratory complications. Some patients survive into adulthood, but exact data are lacking. The severity of the disease is a direct consequence of the global deficiency in NADH-cytochrome b5 reductase activity that characterizes this type of disorder [9].

As both types can present with cyanosis at birth, distinguishing between them clinically can be difficult until differences in motor and cognitive development become apparent, typically between six and nine months of age [2].

### Genetics

Of the 205 cases included in our review, 189 provided information on the genotype. Eighty-one unique *CYB5R3* (NM_000398.7; GRCh38) variants were reported (see Table 2).

**Table 2:** Unique CYB5R3 (NM_000398.7; GRCh38) variants reported in this study

CYB5R3 variant (NM_000398.7)		Variant effect	ACMG class	Zygosity	CYB5R3 variant (NM_000398.7)		Variant effect	RCM type	Case number
c.22-2A > G	NA	splice	LP	homozygous				II	194
c.82C > T	p.(Gln28*)	nonsense	P	compound heterozygous	**c.757 G > A**	p.(Val253Met)	missense	I	157
c.103A > C	p.(Thr35Pro)	missense	P	homozygous				I	28
c.129C > A	p.(Tyr43*)	nonsense	P	compound heterozygous	**c.287C > A**	p.(Pro96His)	missense	II	189
c.136C > T	p.(Arg46Trp)	missense	LP	compound heterozygous	**c.757 G > A**	p.(Val253Met)	missense	I	158
c.148C > T	p.(Arg50Trp)	missense	P	homozygous				I	114, 115
				compound heterozygous	**c.535 G > A**	p.(Ala179Thr)	missense	I	103, 109
c.149 G > A	p.(Arg50Gln)	missense	P	homozygous				I	98, 99
				compound heterozygous	**c.194C > T**	p.(Pro65Leu)	missense	I	174
c.153+1 G > A	exon 2 deletion	splice	P	homozygous				II	151
c.162C > G	p.(Ser54Arg)	missense	LP	homozygous				I	141
c.172C > T	p.(Arg58Trp)	missense	P	homozygous				I	131, 132
c.173 G > A	p.(Arg58Gln)	missense	P	homozygous				I	11, 25, 195, 196, 198
				compound heterozygous	**c.226 G > A**	p.(Gly76Ser)	missense	I	104
c.173 G > C	p.(Arg58Pro)	missense	P	compound heterozygous	**c.226 G > A**	p.(Gly76Ser)	missense	II	137
					**c.563_565del**	p.(Leu188del)	in-frame deletion	II	138
c.175C > T	p.(Arg59Cys)	missense	LP	homozygous				I	15
c.176 G > A	p.(Arg59His)	missense	LP	homozygous				I	30
c.181C > T	p.(Arg61Cys)	missense	P	homozygous				I	32, 59
c.182 G > A	p.(Arg61His)	missense	LP	homozygous				I	130
c.190C > G	p.(Leu64Val)	missense	LP	homozygous				I	19, 20, 21,24
c.194C > T	p.(Pro65Leu)	missense	LP	compound heterozygous	**c.149 G > A**	p.(Arg50Gln)	missense	I	174
c.218T > C	p.(Leu73Pro)	missense	LP	homozygous				I	135, 186
c.226 G > A	p.(Gly76Ser)	missense	P	homozygous				II	42, 119, 120
				compound heterozygous	**c.173 G > A**	p.(Arg58Gln)	missense	I	104
					**c.173 G > C**	p.(Arg58Pro)	missense	II	137
					**c.392T > C**	p.(Leu131Pro)	missense	II	148
					**c.827C > T**	p.(Pro276Leu)	missense	I	162
c.229C > T	p.(Gln77*)	nonsense	P	homozygous				II	40
				compound heterozygous	**c.478C > T**	p.(Arg160*)	nonsense	II	183
c.250C > T	p.(Arg84*)	nonsense	P	homozygous				II	184
c.254T > G	p.(Ile85Ser)	missense	LP	homozygous				I	160
c.274C > T	p.(Arg92Trp)	missense	LP	homozygous				II	13, 14,45, 46
c.287C > A	p.(Pro96His)	missense	P	compound heterozygous	**c.129C > A**	p.(Tyr43*)	nonsense	II	189
c.310 G > T	p.(Gly104Cys)	missense	P	homozygous				I	34
c.316 G > A	p.(Val106Met)	missense	P	homozygous				I	197
				compound heterozygous	**c.655C > T**	p.(Arg219*)	nonsense	I	156
c.332A > T	p.(Lys111Met)	missense	LP	homozygous				II	150
c.334-2A > G	exon 5 deletion	splice	P	compound heterozygous	**c.379A > G**	p.(Met127Val)	missense	II	179
c.334-1 G > A	exon 5 deletion	splice	P	compound heterozygous	**c.716T > G**	p.(Leu239Arg)	missense	I	175
c.352C > T	p.(His118Tyr)	missense	LP	homozygous				I	35, 38
c.379A > G	p.(Met127Val)	missense	P	homozygous				I	23
				compound heterozygous	**c.334-2A > G**	exon 5 deletion	splice	II	179
c.382T > C	p.(Ser128Pro)	missense	P	homozygous				II	201, 202, 203
c.392T > C	p.(Leu131Pro)	missense	P	compound heterozygous	**c.226 G > A**	p.(Gly76Ser)	missense	II	148
c.402 G > C	p.(Met134Ile)	missense	VUS	homozygous				I	55
c.431 G > A	p.(Gly144Asp)	missense	P	homozygous				I	36, 152, 155
				compound heterozygous	**c.871 G > A**	p.(Val291Met)	missense	I	9, 10
c.431 G > T	p.(Gly144Val)	missense	LP	homozygous				I	4
c.433C > T	p.(Pro145Ser)	missense	P	homozygous				I	107, 165
				compound heterozygous	**c.757 G > A**	p.(Val253Met)	missense	I	159
c.446T > C	p.(Leu149Pro)	missense	P	homozygous				I	199, 200
c.463+2T > C	NA	splice	P	homozygous				II	154, 164
c.463+8 G > C	exon 5 deletion	splice	P	homozygous				II	2,3, 149, 190
c.464-2A > C	exon 6 deletion	splice	LP	homozygous				II	187
c.464 G > A	p.(Gly155Glu)	missense	P	homozygous				I	101
c.470T > G	p.(Phe157Cys)	missense	P	homozygous				I	37
				compound heterozygous	**c.757 G > A**	p.(Val253Met)	missense	I	142
c.470T > C	p.(Phe157Ser)	missense	P	homozygous				I	16
c.478C > T	p.(Arg160*)	nonsense	P	compound heterozygous	**c.229C > T**	p.(Gln77*)	nonsense	II	183
c.479 G > C	p.(Arg160Pro)	missense	P	homozygous				I	29, 116
c.514 G > C	p.(Val172Leu)	missense	VUS	homozygous				I	133, 134
c.517_525del	p.(Lys173_Val175del)	in-frame deletion	LP	homozygous				II	124
c.529A > G	p.(Met177Val)	missense	P	homozygous				I	111, 113
c.531 G > A	p.(Met177Ile)	missense	P	homozygous				I	110
c.533T > C	p.(Ile178Thr)	missense	P	homozygous				I	102
c.535 G > A	p.(Ala179Thr)	missense	P	homozygous				I	1, 12, 105, 106, 112, 118, 139, 140, 176, 177
				compound heterozygous	**c.148C > T**	p.(Arg50Trp)	missense	I	103, 109
c.536C > T	p.(Ala179Val)	missense	P	homozygous				I	185
c.563_565del	p.(Leu188del)	in-frame deletion	LP	compound heterozygous	**c.173 G > C**	p.(Arg58Pro)	missense	II	138
c.571A > T	p.(Ile191Phe)	missense	LP	homozygous				I	6, 7, 8
c.574C > T	p.(Arg192Cys)	missense	P	homozygous				I	22,27,31,33, 47–54
c.605_606del	p.(Thr202Serfs*19)	frameshift	P	homozygous				II	41
c.610T > C	p.(Cys204Arg)	missense	P	compound heterozygous	**c.815_817del**	p.(Met273del)	in-frame deletion	II	147, 192
c.611 G > A	p.(Cys204Tyr)	missense	P	homozygous				I	180, 181, 182
				compound heterozygous	**c.708 G > A**	p.(Trp236*)	nonsense	II	143, 144, 145
					**c.906A > G**	p.(*302Trpext*42)	stop loss	I	5
c.637 G > A	p.(Glu213Lys)	missense	VUS	homozygous				I	188
c.647T > C	p.(Ile216Thr)	missense	P	compound heterozygous	**c.757 G > A**	p.(Val253Met)	missense	I	168
c.653T > C	p.(Leu218Pro)	missense	P	homozygous				I	26, 146
c.655C > T	p.(Arg219*)	nonsense	P	homozygous				II	191
				compound heterozygous	**c.316 G > A**	p.(Val106Met)	missense	I	156
c.708 G > A	p.(Trp236*)	nonsense	P	homozygous				II	121
				compound heterozygous	**c.611 G > A**	p.(Cys204Tyr)	missense	II	143, 144, 145
c.713C > T	p.(Thr238Met)	missense	P	homozygous				I	117
c.716T > G	p.(Leu239Arg)	missense	P	compound heterozygous	**c.334-1 G > A**	exon 5 deletion	splice	I	175
c.721A > G	p.(Arg241Gly)	missense	VUS	homozygous				II	166
c.734-2del	NA	splice	P	homozygous				II	17
c.757 G > A	p.(Val253Met)	missense	P	homozygous				I	108, 161, 170, 171, 172, 173, 178
				compound heterozygous	**c.82C > T**	p.(Gln28*)	nonsense	I	157
					**c.136C > T**	p.(Arg46Trp)	missense	I	158
					**c.433C > T**	p.(Pro145Ser)	missense	I	159
					**c.470T > G**	p.(Phe157Cys)	missense	I	142
					**c.647T > C**	p.(Ile216Thr)	missense	I	168
c.766_768del	p.(Glu256del)	in-frame deletion	P	homozygous				II	39
				compound heterozygous	**c.875 G > A**	p.(Gly292Asp)	missense	I	169
c.802_804del	p.(Glu268del)	in-frame deletion	VUS	homozygous				I	125, 126
c.806C > T	p.(Pro269Leu)	missense	LP	homozygous				I	60–97, Galeeva 2013
c.815_817del	p.(Met273del)	in-frame deletion	P	compound heterozygous	**c.610T > C**	p.(Cys204Arg)	missense	II	147, 192
c.827C > T	p.(Pro276Leu)	missense	P	compound heterozygous	**c.226 G > A**	p.(Gly76Ser)	missense	I	162
c.830dup	p.(Pro278Thrfs*91)	frameshift	P	homozygous				II	18
c.871 G > A	p.(Val291Met)	missense	VUS	compound heterozygous	**c.431 G > A**	p.(Gly144Asp)	missense	I	9, 10
c.875 G > A	p.(Gly292Asp)	missense	P	compound heterozygous	**c.766_768del**	p.(Glu256del)	in-frame deletion	I	169
c.882_884delinsAA	p.(Thr295Argfs*54)	frameshift	P	homozygous				II	163
c.895_897del	p.(Phe299del)	in-frame deletion	LP	homozygous				II	193
c.906A > G	p.(*302Trpext*42)	stop loss	LP	compound heterozygous	c.611 G > A	p.(Cys204Tyr)	missense	I	5

ACMG: American College of Medical Genetics and Genomics; LP: likely pathogenic; NA: not availableP: pathogenic; RCM: recessive congenital methemoglobinemia; VUS: variant of unknown significance

Of these, 75 variants are classified as pathogenic or likely pathogenic, and six as variants of unknown significance (VUS) (see Supplementary Table A). All variants are rare in the general population (occurring at a frequency of < 0.05% in gnomAD), except for the c.637G > A, p.(Glu213Lys) variant, which was classified as a VUS. Some variants are recurrent, occurring in ≥ 2 unrelated families. These include c.535 G > A, p. (Ala179Thr) (*n* = 12, including our case), c.757 G > A, p.(Val253Met) (*n* = 12), c.226 G > A, p.(Gly76Ser) (*n* = 7) and c.173 G > A, p.(Arg58Gln) (*n* = 6). While most of the 81 variants are missense variants (*n* = 56), splice site variants (*n* = 8), nonsense variants (*n* = 7), in-frame deletions (*n* = 6), frameshift variants (*n* = 3) and one stop-loss variant are also detected. These variants are spread throughout the entire gene and affect the different protein domains. Biallelic (likely) pathogenic nonsense, frameshift, and splice site variants invariably lead to type II methemoglobinemia. In all other cases, genotype-phenotype correlation is based on the specific variant.

Previous reviews have associated certain pathogenic variants with both type I and II RCM.

The c.757 G > A, p.(Val253Met) missense variant is a well-known recurrent variant that causes type I methemoglobinemia when present in the homozygous or compound heterozygous state (cases 108, 142, 157, 158, 159, 161, 168, 170–173, 178). However, Kugler et al. [15] describe a 49-year-old patient who was homozygous for the c.757 G > A, p.(Val253Met) variant, presenting with cyanosis, amaurosis, and severe neurodevelopmental delay from infancy. Methemoglobinemia was diagnosed in adulthood based on MetHb levels of 7.5% and cytochrome b5 reductase activity of 20% in erythrocytes. The condition was classified as RCM type II. However, confirmation of this clinical diagnosis through measurement of cytochrome b5 reductase activity in lymphocytes or other non-erythroid cell lines is lacking. Detailed clinical data on the neurological phenotype or the presence or absence of more specific clinical findings, such as microcephaly or motor symptoms, are not provided. Conversely, they report amaurosis, which is not a recognised feature of type II methemoglobinemia. Hereditary amaurosis has been associated with neurological and neurodevelopmental symptoms [16]. In conclusion, it is plausible that this patient has type I methemoglobinemia associated with psychomotor delay of a separate etiology, as previously hypothesized by Ewenczyk et al. [14].

The c.226 G > A p.(Gly76Ser) missense variant has been associated with both types I and type II of methemoglobinemia in previous reviews [17, 18]. However, we observed that type II methemoglobinemia is associated with homozygous variants, whereas type I is present in cases of compound heterozygosity involving c.226 G > A p.(Gly76Ser) and c.173 G > A, p.(Arg58Gln) or c.827C > T, p.(Pro276Leu). Similar results have been observed for multiple other variants, highlighting that the resulting phenotype is determined by the specific combination of the two allelic variants and the final enzyme conformation. This is an important consideration when evaluating compound heterozygous variants [14].

Thoroughly evaluating the original descriptions of the c.562_564del, p.(Leu188del) and c.726_729del, p.(Glu266del) variants revealed that the previously reported ambiguous results were incorrect. These variants are specific to methemoglobinemia types II and type I, respectively.

In conclusion, the phenotype cannot be reliably predicted from the variant type or protein localization. However, when considered in combination with their allelic variants in compound heterozygous cases, the described pathogenic variants are specific to either type I or type II methaemoglobinaemia.

### Diagnosis

RCM is diagnosed by detecting elevated metHb levels, decreased cytochrome b5 reductase activity and bi-allelic pathogenic variants in the *CYB5R* gene [3]. A metHb percentage of more than 10% should raise concerns about hereditary methemoglobinemia. In this review, we estimated the metHb percentage to be greater than 10% in 92% [95% CI 87%-96%] of cases. The estimated average metHb percentage was 22.3% [95% CI 19.4–25.3] for type I and 28.6% [95% CI 24.5–32.6] for type II. The metHb percentage was statistically higher in type II than in type I (*p* = 0.007) (Table 1).

Reduced erythrocyte cytochrome b5 reductase activity was observed in around 60% of cases. However, enzyme activity was only tested in leukocytes, platelets, and/or fibroblasts in 20 out of 205 cases. When measured in erythroid and non-erythroid cells, enzyme activity can serve as a valuable diagnostic test to distinguish between types I and type II. Therefore, when available, enzyme assays should be performed in erythroid and non-erythroid cells, such as lymphocytes or fibroblasts [19]. We found that the estimated mean erythrocyte metHb reductase activity was significantly higher in type I than in type II (7.17 [95% CI 4.74–9.60] U/g Hb and 4.02 [95% CI 1.39–6.65] U/g Hb respectively; *p* < 0.001) (Table 1). However, the availability of non-erythroid enzyme activity testing is limited, partly due to laboratory-specific reference values, which restricts its application in clinical practice. It is currently impossible to propose universal reference values for cytochrome b5 enzymatic activity. We can conclude that the residual enzyme activity in erythrocytes is usually less than 20% of normal levels. In newborns, cytochrome b5 reductase activity is 60% of the adult level. Adult levels are reached by 2- 3 months of age [20]. Due to lower levels of cytochrome b5 reductase activity, neonates are at greater risk of developing methemoglobinemia [21]. Additionally, hemoglobin F levels are higher in neonates and are more easily oxidized to metHb than hemoglobin A in adults.

Molecular genetic analysis of the *CYB5R3* gene can confirm the diagnosis of hereditary methemoglobinemia. Based on a review of reported disease-associated variants, diagnostic molecular testing should include DNA sequencing of all *CYB5R3* gene exons, including the exon-intron boundaries. Direct sequencing of the full *CYB5R3* gene was performed in the majority of cases (134/182, 74%). However, exome sequencing is becoming increasingly prevalent, as evidenced by more recent studies (9/182, 5%). Specifically for type II RCM, most authors applied a targeted sequencing approach (36/37, 97%) (Table 3). Given the predominantly neurological symptoms of type II RCM, we recommend including *CYB5R3* in NGS panels that target neurodevelopmental disorders.

**Table 3:** Type of genetic analysis

	Type I	Type II
	n = 145	n = 37
	k = 36	k = 20
Genetic analysis		
Direct sequencing CYB5R3	98 (67.5)	36 (97.3)
Exome sequencing	8 (5.5)	1 (2.7)
MLPA for c.806C > T mutation	38 (26.2)	0
NGS gene panel	1 (0.7)	0

Data presented as n (%); *n* = number of observations; k = number of studies

**Fig. 2:**
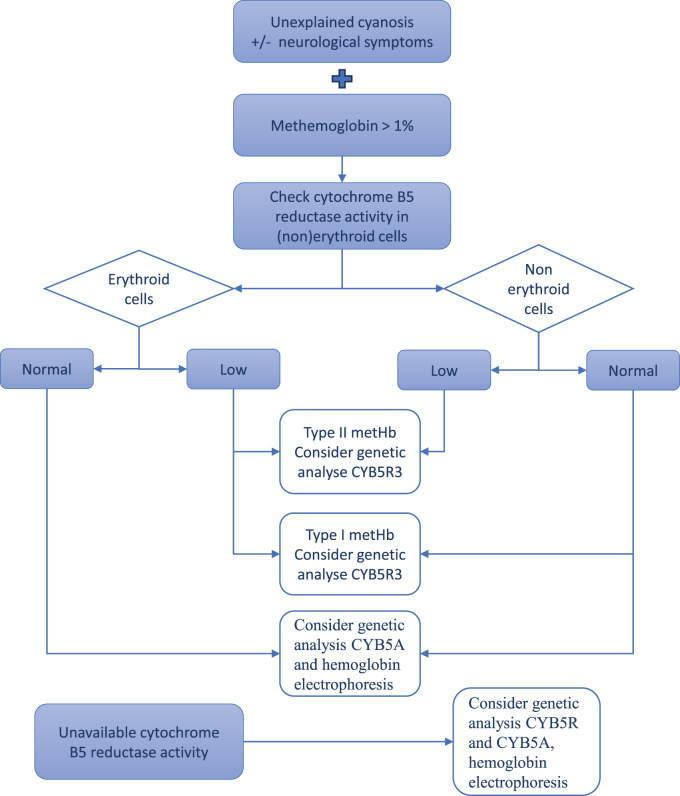
A proposal for a diagnostic flow chart for methemoglobinemia (> 1%)

If congenital methemoglobinemia is suspected, an important differential diagnosis to consider is autosomal dominant methemoglobinemia, also known as Hemoglobin M disease. Hemoglobin M can be detected using hemoglobin electrophoresis. This disease results from mutations in the alpha-globin (HBA1 and HBA2), beta-globin (HBB), or gamma-globin (HBG1 and HBG2) genes [21]. Lastly, a rare deficiency of the electron acceptor cytochrome b5, known as methemoglobinemia type IV, is caused by mutations in the cytochrome b5 gene (*CYB5A*) [22]. Therefore, these alternative causes of methemoglobinemia should be ruled out using hemoglobin electrophoresis and genetic analysis of the *CYB5A* gene [3, 22]. In both disorders, neurological features are absent. A proposed diagnostic flowchart is provided in Fig. 2.

### Treatment

**Fig. 3:**
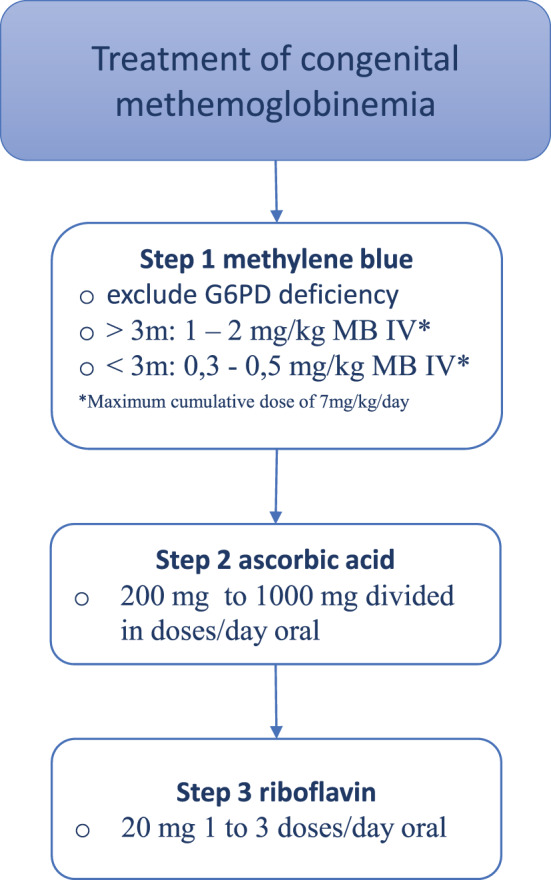
Treatment options for reducing methemoglobin levels

Treatment for autosomal recessive methemoglobinemia is usually only recommended for symptomatic patients with methemoglobin levels above 10% [4]. There are three treatment options (see Fig. 3). Aim of treatment is reduction of methemoglobin levels under treatment level (< 10%). This review reports on the treatment of 32 cases of type I RCM and 14 cases of type II RCM. In 26 cases methemoglobin levels after treatment are reported. On average treatment with mono- or combination therapy decreases methemoglobin levels (%) to 18.7 [8.6 - 37.4] in type I and 17.1 [1.6 - 34] in type II.

The first-line therapy for symptomatic patients without G6PD deficiency is methylene blue (MB) [21]. MB is reduced to leuco-MB by flavin reductase in erythrocytes, which acts as an electron donor to transfer electrons from NADPH to reduce Fe^3+^ to Fe^2+^ [23]. NADPH is generated by G6PD. Therefore, MB is counterproductive in cases of G6PD deficiency and can cause severe hemolysis [24]. The initial dose is 1–2 mg/kg of MB in a 1% solution in 0.9% saline, administered intravenously over five minutes. If there is no response after 30 minutes, the process should be repeated at a dose of 1 mg/kg, up to a maximum cumulative dose of 5.5-7 mg/kg/day [21]. The European Medicines Agency (EMA) supports this dosage for adults and children over three months. For children younger than three months, a dosage of 0.3–0.5 mg/kg intravenously is recommended. Of the 46 cases with information, only 11 involved treatment with oral or intravenous MB, which caused a decline in metHb levels (Table 4). In all reported type I cases (4) methemoglobin levels reached target after MB monotherapy. Occasionally, 100 to 300 mg of oral MB per day can be administered, adjusted according to metHb levels [3]. The estimated proportion of cases with MB in type I was 40% [95% CI 20%-59%] and 15% [95% CI 0%-34%] in type II. There was no statistically significant difference between the two types (*p* = 0.12) (Table 1).

**Table 4:** Patients with congenital metHb divided in age-groups, diagnostic tests, and therapy

	Type I						Type II					
		Diagnostic test		Therapy				Diagnostic test		Therapy		
**Age (y)**	**Cases**	**MetHb%**^**1**^	**eMBR activity**	Aa %*	Mb %*	Riboflavin %*	**Cases**	**MetHb%**^**1**^	**eMBR activity**	Aa %*	Mb %*	Riboflavin %*
			**(U/g Hb)**^**1**^						**(U/g Hb)**^**1**^			
0–4	29	21,9 (27)	6,8 (19)	24 (7)	14 (4)		27	32,1 (24)	7,8 (14)	41 (11)	11 (3)	4 (1)
5–9	17	16,9 (16)	11,2 (7)	18 (3)	6 (1)		6	31,5 (6)	9,2 (5)	33 (2)		
10–14	20	18 (20)	15,4 (8)	10 (2)			4	19,9 (2)	7,5 (2)			
15–19	26	22,6 (26)	13,2 (8)	15 (4)	4 (1)							
20–24	11	24,8 (10)	15,8 (5)	45 (5)			1	10,7 (1)				
25–29	9	33,6 (9)	14,7 (6)	44 (4)								
30–34	4	25,5 (4)	15,2 (4)									
35–39	4	17,1 (4)	16,8 (1)		25 (1)	25 (1)						
40–44	5	21,1 (5)	14,4 (4)	20 (1)								
45–49	3	20,4 (3)	8,1 (2)									
50–54	2	13,4 (2)	13 (2)									
55–59	2	13,9 (2)		50 (1)								
60–64	2	34,5 (2)			50 (1)	50 (1)						
65–69	1	26,2 (1)		100 (1)								
70–74	0											
75–79	2	30,1 (2)										
80–85	0											

eMBR activity = erythrocyte metHb reductase activity, Aa = Ascorbic acid, Mb = Methylene blue

percentage cases of the total cases in this agegroup, in brackets number of cases

average numbers, in brackets number of cases tested

Ascorbic acid (vitamin C) can be used as an alternative or alongside MB [3, 25]. Ascorbic acid facilitates the reduction of metHb by acting as a cofactor for NADP reductase [25]. Various doses ranging from 200 mg to 1000 mg orally per day, taken in divided doses, have been reported in the literature [2, 3]. It takes up to 24 hours to lower metHb levels. Of the cases with information, 41 out of 46 cases were treated with vitamin C, either with or without MB (see Table 4). In 5 of 8 reported type I and 1 of 4 reported type II cases, methemoglobin levels reached target after vitamin C monotherapy. In combination with MB, all reported type I (7) and all reported type II cases (2) reached target methemoglobin levels. Chronic administration may lead to the formation of sodium oxalate nephrolithiasis [3]. The estimated proportion of cases involving ascorbic acid was 86% [95% CI 73%-99%] for type I and 93% [95% CI 80%-100%] for type II. There was no statistically significant difference between the two types (*p* = 0.51) (Table 1).

The final therapeutic option is riboflavin (vitamin B2). Riboflavin acts as an electron acceptor via the nicotinamide adenine dinucleotide-flavin reductase system, accelerating the reduction of metHb [3, 23]. However, the available data on the use of riboflavin in hereditary methemoglobinemia are limited. In the reported cases, riboflavin is administered at 20–30 mg/day or 20 mg three times daily. In this review, only three patients were started on riboflavin therapy (see Table 4). One reported type II case reached methemoglobin target level with combination therapy of riboflavin and vitamin C.

There is no effective treatment for the neurological symptoms [2]. Most patients in this review underwent treatment involving the administration of ascorbic acid, either as a standalone therapy or in combination with methylene blue. In both type I and II, treatment with MB monotherapy or combination with vitamin C leads in all reported cases to methemoglobin levels beneath target.

## Limitations

We acknowledge that this systematic review has several limitations. Firstly, the clinical details of patients with congenital methemoglobinemia described above are limited. This is due to the differing focus of the articles, as well as their heterogeneity, which makes quality assessment difficult. Secondly, type I congenital methemoglobinemia may be grossly underdiagnosed, as most patients present with mild or no symptoms of cyanosis. Thirdly, there is a lack of standard reference ranges for cytochrome b5 reductase enzyme activity in erythroid and non-erythroid cells. Data on non-erythroid enzyme assays are minimal, and testing is impractical in standard clinical practice due to limited availability. Finally, there is a lack of exact data on the dosage and duration of therapy with methylene blue, ascorbic acid, and/or riboflavin, necessitating further investigation.

## Conclusion

Recessive congenital methemoglobinemia presents in two distinct clinical forms. Type I is generally benign and is characterized primarily by cyanosis, and associated with a normal life expectancy. In contrast, type II is more severe being associated with significant neurological impairment, developmental delay, microcephaly, and a significantly reduced life expectancy. It typically results in death during the first decade of life.

Diagnosis involves measuring metHb levels and assessing cytochrome b5 reductase activity in erythroid cells and, if available, in non-erythroid cells. This also involves hemoglobin electrophoresis and confirmation through molecular analysis of the *CYB5R* gene (see Fig. 2).

Thanks to the advances in molecular genetics and the widespread adoption of next-generation sequencing techniques in clinical practice, genetic testing is now considered the gold standard for diagnosing hereditary methemoglobinemia [3]. A molecular diagnosis can confirm a clinical diagnosis and provide information from a clinical outcome perspective because the described pathogenic variants, when considered in combination with their allelic variants in compound heterozygous cases, are specific to either type I or type II methemoglobinemia. It is beneficial to differentiate between types I and II when counselling young patients, as the phenotype may not be clinically apparent and the neurological examination may be normal in the first months of life for patients with type II [14]. Furthermore, identifying the genotype enables families to make informed decisions regarding reproductive strategies for future pregnancies where type II methemoglobinemia is expected, such as preconception genetic testing or prenatal diagnosis This critical review of genetic variants, which provides an exhaustive and up-to-date list of variants, which will help counsel families with recessive congenital methemoglobinemia.

Treatment options for reducing methemoglobin levels include methylene blue, ascorbic acid, and riboflavin (see Fig. 3). However, there is currently no effective treatment for the neurological symptoms associated with type II methemoglobinemia. Distinguishing between types I and type II is crucial for prognosis and management, but this can be challenging without molecular and enzyme assays.

## Electronic supplementary material

Below is the link to the electronic supplementary material.

Supplementary Material 1

## Data Availability

All data generated or analyzed during this study are included in this published article [and its supplementary information files].

## Abbreviations

metHb: Methemoglobin
Hb: Hemoglobin
Fe^3+^: Iron in ferric state
Fe^2+^: Iron in ferrous state
G6PD: Glucose-6-phosphate-dehydrogenase
MB: Methylene blue
EMA: European Medicines Agency
RCM: Recessive congenital methemoglobinemia
VUS: Variant of unknown significance
IPD: Individual Participant Data
GEE: Generalized estimating equations
