# Correction: Recessive congenital methemoglobinemia: a systematic review of reported cases

**DOI:** 10.1186/s13023-026-04372-9

**Published:** 2026-06-05

**Authors:** Julie Neven, Tessi Beyltjens, Marije Meuwissen, Barbara De Bisschop, Jason Bouziotis, Machiel van den Akker

**Affiliations:** 1 Department of Pediatrics, University Hospital Antwerp, University of Antwerp, Drie Eikenstraat 655, Edegem, 2650 Belgium; 2 Center of Medical Genetics, University Hospital Antwerp/ University of Antwerp, Prins Boudewijnlaan 43, Edegem, 2650 Belgium; 3 Department of Neonatology, ZAS Middelheim Hospital, Lindendreef 1, Antwerp, 2020 Belgium; 4 Center for Medical Genetics Ghent, Ghent University Hospital, Ghent, 9000 Belgium; 5 Clinical Trial Center (CTC), CRC Antwerp, Antwerp University Hospital University of Antwerp, Edegem, Belgium; 6 Faculty of Medicine and Health Sciences, University of Antwerp, Antwerp, Belgium; 7 Department of Pediatric Hematology, ZAS Queen Paola Children’s Hospital, Lindendreef 1, Antwerp, 2020 Belgium; 8 Pediatric Hematology Oncology, University Hospital Antwerp, DrieEikenstraat 655, Edegem, Antwerp, 2650 Belgium

**Correction: Orphanet Journal of Rare Diseases (2026) 21:89**

10.1186/s13023-026-04215-7

Following publication of the original article [1], we have been notified about several incorrectly published body text parts.

Author name was incorrectly published. It is now:

Beyltjens E. C. Meuwissen^2^.

It should be: Marije Meuwissen^2^.

Incorrect body text parts were:

Information of the cases is summarized in Tables 1 (meta-analysis results of both binary and continuous variables), 2 (diagnostic tests and therapy), 3 (unique CYB5R3 variants) and 4 (type of genetic analysis). One supplementary table is available online: Table A (patient data).

It takes up to 24 hours to lower metHb levels. Of the cases with information, 41 out of 46 cases were treated with vitamin C, either with or without MB (see Table 3).

In the reported cases, riboflavin is administered at 20–30 mg/day or 20 mg three times daily. In this review, only three patients were started on riboflavin therapy (see Table 2).

They should be:

Information of the cases is summarized in Tables 1 (meta-analysis results of both binary and continuous variables), 2 (unique CYB5R3 variants), 3 (type of genetic analysis), and 4 (diagnostic tests and therapy). One supplementary table is available online: Table A (patient data).

It takes up to 24 h to lower metHb levels. Of the cases with information, 41 out of 46 cases were treated with vitamin C, either with or without MB (see Table 4).

In the reported cases, riboflavin is administered at 20–30 mg/day or 20 mg three times daily. In this review, only three patients were started on riboflavin therapy (see Table 4).

Tables 2 and 3 were switched and published incorrectly.

The original article was updated.
